# The roles of Syx5 in Golgi morphology and Rhodopsin transport in *Drosophila* photoreceptors

**DOI:** 10.1242/bio.020958

**Published:** 2016-09-02

**Authors:** Takunori Satoh, Yuri Nakamura, Akiko K. Satoh

**Affiliations:** Division of Life Science, Graduate School of Integral Arts and Science, Hiroshima University, 1-7-1, Kagamiyama, Higashi-hiroshima 739-8521, Japan

**Keywords:** Syx5, Rhodopsin, ER, Golgi, Vesicle cluster

## Abstract

SNAREs (SNAP receptors) are the key components of protein complexes that drive membrane fusion. Here, we report the function of a SNARE, Syntaxin 5 (Syx5), in the development of photoreceptors in *Drosophila*. In wild-type photoreceptors, Syx5 localizes to *cis-*Golgi, along with *cis-*Golgi markers: Rab1 and GM130. We observed that Syx5-deficient photoreceptors show notable accumulation of these *cis-*Golgi markers accompanying drastic accumulation of vesicles between endoplasmic reticulum (ER) and Golgi cisternae. Extensive analysis of Rh1 (rhodopsin 1) trafficking revealed that in Syx5-deficient photoreceptors, Rh1 is exported from the ER with normal kinetics, retained in the *cis*-Golgi region along with GM130 for a prolonged period, and then subsequently degraded presumably by endoplasmic reticulum-associated protein degradation (ERAD) after retrieval to the ER. Unlike our previous report of Rab6-deficient photoreceptors – where two apical transport pathways are specifically inhibited – vesicle transport pathways to all plasma membrane domains are inhibited in Syx5-deficient photoreceptors, implying that Rab6 and Syx5 are acting in different steps of intra-Golgi transport. These results indicate that Syx5 is crucial for membrane protein transport, presumably during ER-derived vesicle fusion to form *cis-*Golgi cisternae.

## Abstract (precis)

**Summary:** Unlike Rab6-deficiency which affects only apical transport pathways, Syx5-deficiency inhibits all of polarized transport pathways, implying that these two genes are working in distinct stages of intra-Golgi transport.

## INTRODUCTION

In late pupal *Drosophila* photoreceptors, massive biosynthetic membrane traffic delivers rhodopsin to the rhabdomeres (Kumar and Ready, 1995; Satoh et al., 2005). Therefore, an assay for rhodopsin accumulation in late pupal rhabdomeres offers a sensitive method to identify the genes involved in biosynthetic membrane transport. In a previous study, we screened P-element/piggyBac insertion lines by visualizing Rh1, using Arrestin2::GFP and a water immersion technique, this led to the identification of several lines which failed to accumulate Rh1 at the rhabdomeres (Satoh et al., 2013). In two of these lines, P-elements were inserted in the 5′-UTR of *Syx5*, which encodes a SNARE protein.

SNAREs are the key components of protein complexes that drive membrane fusion (Hong and Lev, 2014; Jahn and Scheller, 2006). SNAREs seem to mediate membrane fusion in all the trafficking steps of the secretory pathway, except the fusion events in mitochondria and peroxisomes. Recent studies have identified two SNARE proteins, Sec22 and Gos28, which play essential roles in transporting Rh1 to the rhabdomeres (Rosenbaum et al., 2014; Zhao et al., 2015). An extensive Rh1 transport assay revealed that Gos28 is involved in intra-Golgi transport downstream of alpha-mannosidase-II in *medial-*Golgi (Rosenbaum et al., 2014). It was also reported that Gos28 mutation affects the modification and transport of Rh1, but not of other proteins including TRP and TRPL, which form the rhabdomeric channels for phototransduction (Niemeyer et al., 1996). In Sec22-deficient photoreceptors, the Rh1 transport is also defective; however, the step at which the Rh1 transport is inhibited, or its effect on other rhabdomeric, stalk or basolateral membrane proteins, has not been described yet. The similarity of morphological defects on ER between Syx5 and Sec22 mutations, and biochemical data indicating Syx5 binding property to Sec22, suggested that Syx5 also functions on Rh1 transport (Zhao et al., 2015).

In the present study, we investigated in detail the effects of Rh1 transport in Syx5-deficient photoreceptors. Our results revealed that Syx5 functions in early Golgi transport, probably in the fusion of COPII vesicles to *cis-*Golgi cisternae. Unlike Rab6-deficiency that only affects the two apical transport pathways, Syx5-deficiency inhibits the transport of membrane proteins destined for any of the plasma membrane domains, implying that these two genes required for intra-Golgi transport are acting in distinct stages.

## RESULTS

### *Syx5* gene is essential for Rh1, TRP, Na^+^K^+^ATPase and Eys transports in fly photoreceptors

**Fig. 1.**
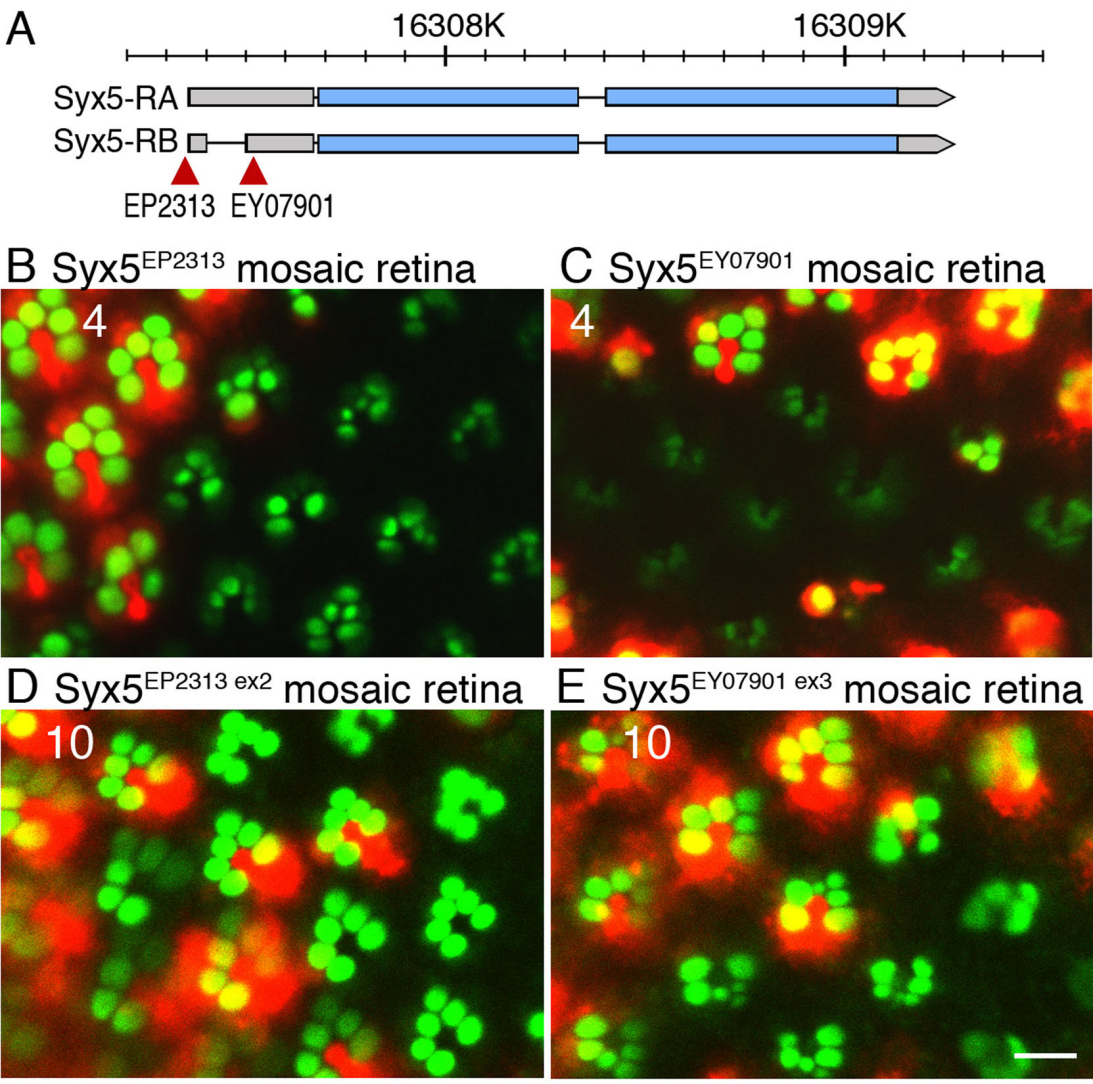
**Failure of Rh1 accumulation on the rhabdomeres in two insertional mutants of *Syx5*.** (A) Schematic view of *Syx5* gene on the genome. Insertion positions for two mutants are indicated with red triangles. Gray bars represent the mRNA sequences, and the blue bars represent the coding sequences. (B-E) Visualization of endogenous Rh1 by Arrestin2::GFP (green) and wild-type cell marker, RFP (red) in *Syx5 ^EP2313^* (B), *Syx5 ^EY0790^*^1^ (C), *Syx5 ^EP2313 ex2^* (D), and *Syx5 ^EY07901 ex1^* (E) mosaic retinas using a water immersion technique. Scale bar: 5 μm. Numbers of the samples observed were shown in the top left corner of the images.

In a previous study, we identified several mutants that failed to accumulate Rh1 in the rhabdomeres, using retinal mosaic screening of P-element or piggy-Bac inserted lines maintained at stock centers (Satoh et al., 2013). Among them, two lines, KY114446 and KY114472 with P-elements EP2313 and EY07901, respectively, were inserted into the 5′-UTR of *Syx5* gene (Fig. 1A). In *Syx5^EP2313^*, and *Syx5^EY07901^* mosaic retinas, mutant photoreceptors displayed smaller rhabdomeres with weak Arrestin2::GFP signals, as compared to the wild-type photoreceptors (Fig. 1B,C). On excising the P-element, EP2313, the phenotype reverted to wild-type, indicating that reduction of Arrestin2::GFP in the rhabdomeres is caused by the insertion of the P-element, EP2313 (Fig. 1D). However, all the five excision lines of EY07901 displayed low Arrestin2::GFP signals in the rhabdomeres, although the corresponding phenotypes were much weaker compared to the original *Syx5^EY07901^* stocks; one example has been shown in Fig. 1E. These results suggest that the chromosome carrying *Syx5^EY0790^*^1^ might harbor another mutation that also inhibits Rh1 accumulation in the rhabdomeres. Therefore, in our study, we chose the *Syx5^EP2313^ allele* to analyze the function of *Syx5* gene.

**Fig. 2.**
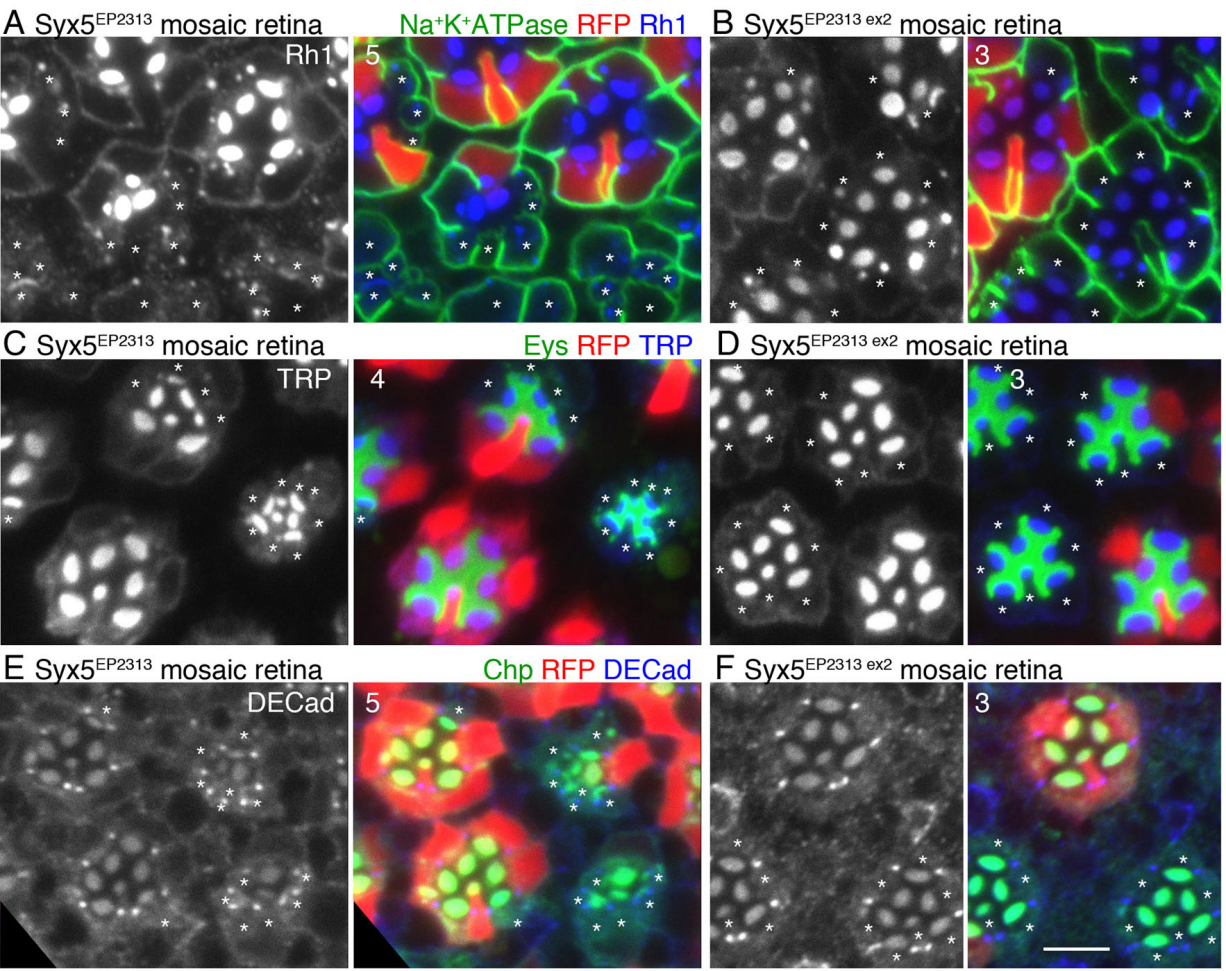
**Levels of membrane proteins, Rh1, Na^+^K^+^ATPase, TRP, and Chp and a secretary protein, Eys, are reduced in Syx5-deficient photoreceptors.** Immunostaining of *Syx5 ^EP2313^* (A,C,E), and *Syx5 ^EP2313 ex2^* (B,D,F) mosaic retinas. RFP (red) marks wild-type cells. Asterisks indicate *Syx5 ^EP2313^*, or *Syx5 ^EP2313 ex2^* homozygous photoreceptors. (A,B) Immunostaining with anti-Rh1 (blue) and anti-Na^+^K^+^ATPase (green) antibodies. (C,D) Immunostaining with anti-TRP (blue) and anti-Eys (green) antibodies. (E,F) Immunostaining with anti-DE-Cad (blue) and anti-Chp (green) antibodies. Scale bar: 5 μm. Numbers of the samples observed were shown in the top-left corner of the composite images.

Immunostaining of *Syx5^EP2313^* mosaic retinas by anti-Rh1 antibody confirmed severe reduction of Rh1 in the rhabdomeres (Fig. 2A). Interestingly, unlike the mutants of genes involved in Rh1 transport, such as Rab11, dRip11, MyoV and Rab6 (Iwanami et al., 2016; Li et al., 2007; Satoh et al., 2005), Rh1 was not detected in the cytoplasm of *Syx5^EP2313^* photoreceptors. First, we observed that in *Syx5^EP2313^* photoreceptors, levels of other rhabdomeric proteins like TRP, Chp (Montell and Rubin, 1989; Reinke et al., 1988) and basolateral membrane protein Na^+^K^+^ATPase (Yasuhara et al., 2000), were severely reduced (Fig. 2). Second, secretion of an extracellular matrix glycoprotein Eys from stalk membrane to the inter rhabdomeral space (IRS) (Husain et al., 2006; Zelhof et al., 2006) is also limited. Like Rh1, TRP, Chp, Na^+^K^+^ATPase, and Eys did not accumulate in the cytoplasm of *Syx5* mutant. Thus, in the *Syx5* mutant, the vesicle transport pathways for the three plasma membrane domains are inhibited. However, adherence junctions formed normally as visualized by DE-Cad (Karagiosis and Ready, 2004) and ommatidia organization was not severely disrupted (Fig. 2E).

Severe reduction in the levels of Rh1, TRP, Chp, Na^+^K^+^ATPase, and Eys observed in *Syx5* mutant photoreceptors reverted to wild-type in *Syx5^EP2313 ex2^* homozygous photoreceptors, indicating that the membrane trafficking defects are caused by the insertion of the P-element, EP2313. However, the rhabdomeres of *Syx5^EP2313 ex2^* homozygotes were still slightly smaller as compared to the wild-type photoreceptors. Sanger sequencing identified about 1400 base-pairs of remnant P-elements in the P-element excision site of *Syx5^EP2313 ex2^*, raising the possibility that *Syx5^EP2313 ex2^* is a weak hypomorphic allele.

### Syx5 localizes to *cis*-Golgi cisternae in fly photoreceptors

In mammalian cells, Syx5 localizes to the ER-derived vesicles, first on the Golgi cisternae and then deep into the Golgi stacks (Hay et al., 1998; Volchuk et al., 2004). Syx5 exists in at least two SNARE subcomplexes: one with membrin, sec22, and bet1 which are involved in fusion between ER-derived vesicles and *cis-*Golgi cisternae; and the other subcomplex with GOS28, Ykt6, and GS15 involved in intra-Golgi transport (Hong and Lev, 2014; Volchuk et al., 2004). This suggests that Syx5 is widely distributed on Golgi stacks.

**Fig. 3.**
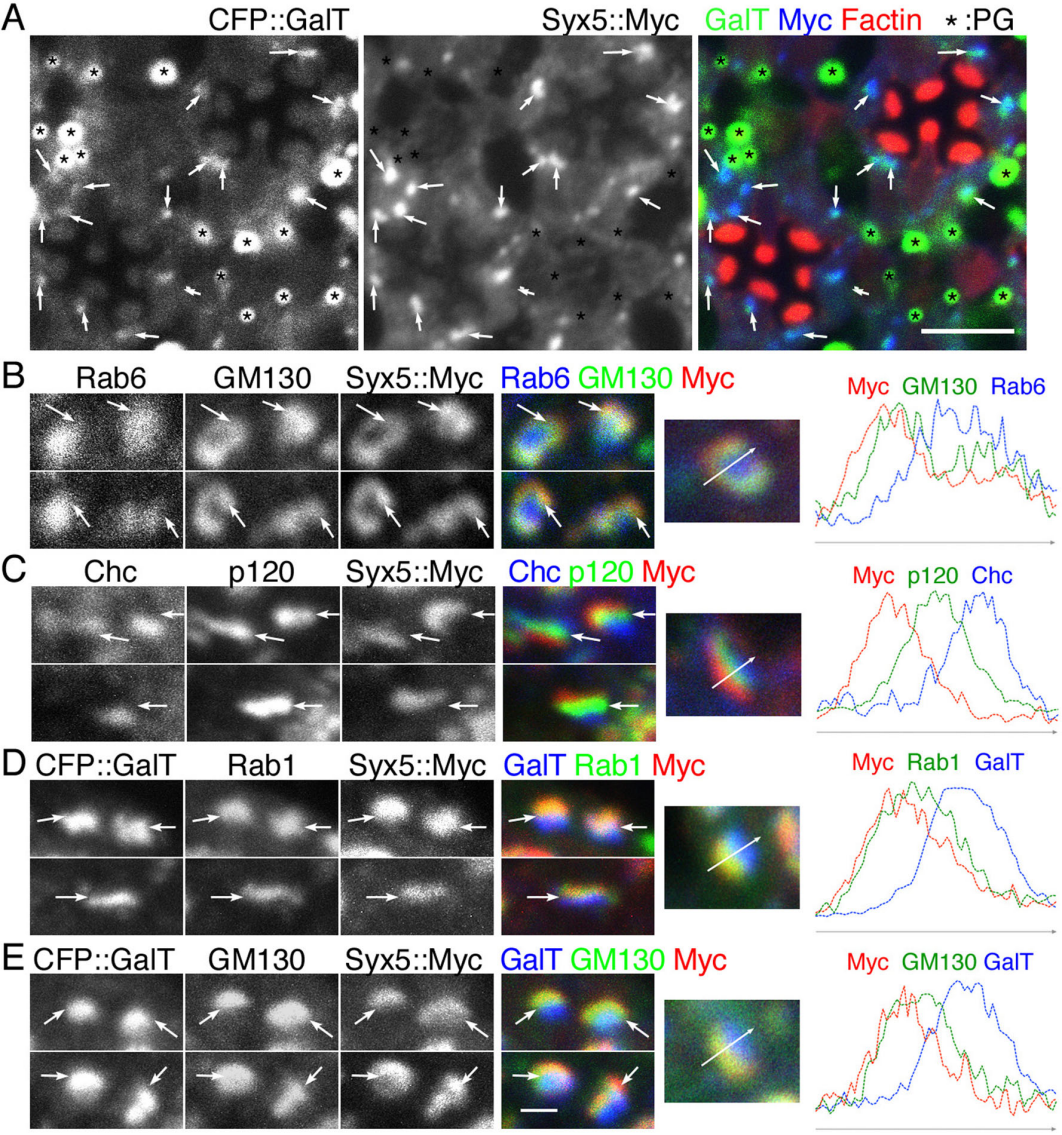
**Syx5 localizes on the *cis* side of Golgi units.** Immunostaining (left panels) of the retinas expressing both CFP::GalT and Syx5::Myc (A,D,E) and Syx5::Myc only (B,C) by GMR-Gal4 driver and plots representing their intensities (right) are shown. (A) CFP::GalT (green), Syx5::Myc (blue) and phalloidin (red). Arrows mark co-localization of CFP::GalT and Syx5::Myc. Asterisks mark auto-fluorescence from pigment granules. (B) Rab6 (blue), GM130 (green), and Syx5::Myc (red). (C) Chc (blue), anti-p120 (green), and Syx5::Myc (red). (D) CFP::GalT (blue), Rab1 (green), and Syx5::Myc (red). (E) CFP::GalT (blue), GM130 (green), and Syx5::Myc (red). Arrows in left panels in B-E indicate the *medial*-Golgi cisternae. Arrows in right panels in B-E indicate the X-axes of the florescent intensity plots. Scale bar: 5 μm in A, 1 μm in B-E. 2 to 4 samples were observed for each of stainings.

In *Drosophila* S2 cells, Syx5 is known to localize on Golgi units (Xu et al., 2002) but its cisternal localization within the Golgi units has not been investigated. Using Syx5::Myc transgenic lines, we examined the localization of Syx5 within the Golgi units in wild-type fly photoreceptors (Norgate et al., 2010). We confirmed Golgi association of Syx5 in late pupal photoreceptors by overexpressing Syx5::Myc and found that it predominantly localizes to the puncta closely associated with the trans-side of Golgi units marked by CFP::GalT (Satoh et al., 2005) (Fig. 3A), unlike Rab6 (another Golgi protein) which localizes to Golgi units and post-Golgi vesicles at the base of the rhabdomeres (Iwanami et al., 2016). Golgi units in photoreceptors are well-developed at the early pupal stages with their width reaching above 1 µm (Satoh et al., 2005). To investigate the association of Syx5 with Golgi-cisternae, we utilized the large Golgi units in photoreceptors of early pupal stages expressing Syx5::Myc (Fig. 3B-E). We observed that Syx5::Myc co-localizes with the *cis-*Golgi marker, GM130, but not with the *medial*-Golgi marker, p120 or the *trans-*Golgi marker, clathrin heavy chain (Chc) (Kametaka et al., 2010; Yamamoto-Hino et al., 2012) (Fig. 3B and 2D). Interestingly, Syx5::Myc signals were slightly shifted further towards the cis-side as compared to the *cis-*Golgi marker, GM130 (Fig. 2B). We also examined Syx5::Myc localization in the photoreceptors expressing both Syx5::Myc and *trans*-Golgi marker GalT::CFP and observed that Syx5::Myc co-localizes very well with *cis-*Golgi marker Rab1, but not with *trans*-Golgi marker GalT::CFP (Satoh et al., 2005) (Fig. 2D,E). These results indicate that Syx5 localizes primarily on Rab1-positive ER-derived vesicles and *cis-*Golgi cisternae in fly photoreceptors.

### Amplification of *cis*-Golgi markers in Syx5-deficient photoreceptors

In a previous study, ectopic expression of ER-resident KDEL receptor in Syx5-deficient fat body cells displayed abnormal localization, suggesting ER membrane malformation (Zhao et al., 2015). Thus, we next determined the localization, and the level of resident proteins in ER and Golgi. Unlike ectopically expressed KDEL receptors in Syx5-deficient fat body cells, endogenous KDEL-containing proteins, calnexin (Cnx) (Rosenbaum et al., 2006), and NinaA (a rhodopsin chaperon) (Colley et al., 1991) were expressed and localized normally in Syx5-deficient photoreceptors (Fig. S1). Therefore, we conclude that the phenotype of ER membrane amplification is not obvious in photoreceptors of late pupal stages.

**Fig. 4.**
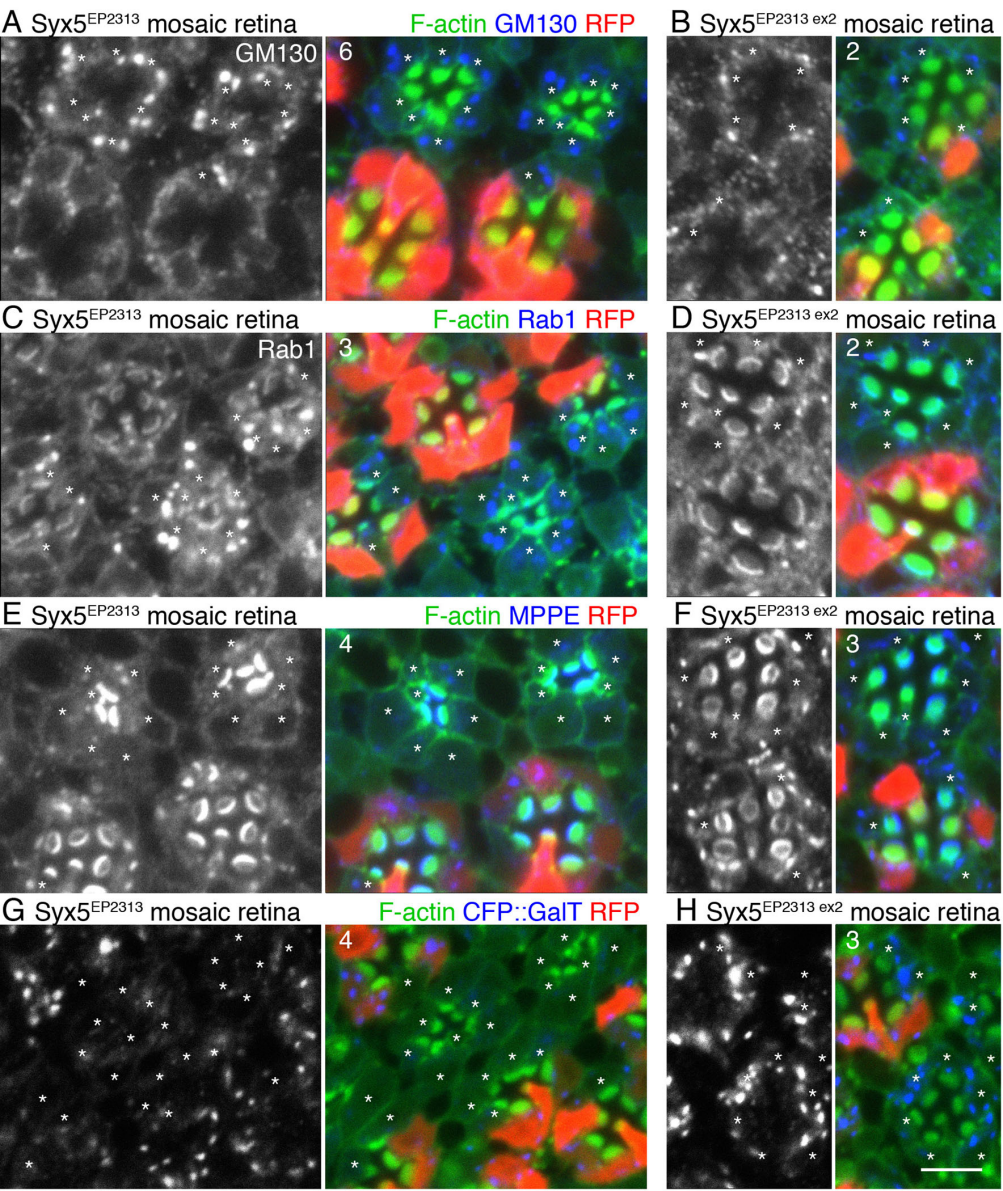
**Marked increase in *cis*-Golgi markers, GM-130, and Rab1, in Syx5- deficient photoreceptors.** *Syx5^EP2313^* (A,C,E,G) or *Syx5^EP2313 ex2^* (B,D,F,H) mosaic retinas were immunostained with one of the following antibodies: anti-GM130 (A,B), anti-Rab1(C,D), anti-MPPE (E,F), or anti-GFP antibody (G,H), along with phalloidin (A-H). RFP (red) marks wild-type cells. Asterisks indicate *Syx5^EP2313^* or *Syx5^EP2313 ex2^* homozygous photoreceptors. In G and H, peripheral photoreceptors in mosaic retinas express GalT::CFP. (A,B) GM130 (blue) and phalloidin (green). (C,D) Rab1 (blue) and phalloidin (green). (E,F) MPPE (blue) and phalloidin (green). (G,H) GFP (blue) and phalloidin (green). Scale bar: 5 μm. Numbers of the samples observed were shown in the top left corner of the composite images.

In contrast to the normal level and distribution pattern of resident ER proteins, staining of peripheral membrane proteins GM130 (on *cis-*Golgi cisternae), and Rab1 (on COPII vesicles) was notably increased in Syx5-deficient photoreceptors (Fig. 4A,B). On the other hand, both ManII-metallophosphoesterase (MPPE) (Cao et al., 2011) and GalT::CFP resident membrane proteins in *medial-* or *trans-*Golgi cisternae, respectively, disappeared. These phenotypes relating to the distribution and amount of Golgi resident proteins were rescued in *Syx5^EP2313 ex2^* photoreceptors.

These results indicate that in Syx5-deficient photoreceptors, fusion of COPII vesicles to *cis-*Golgi cisternae are inhibited, resulting in the accumulation of COPII vesicles and *cis*-Golgi cisternae. Additionally, there is a reduction in the number of *medial-* and *trans-*Golgi cisternae owing to a lack of *cis-*Golgi cisternae that can progressively maturate into *medial*- and *trans*-Golgi cisternae. These phenotypes are distinct from that observed in *Rab6* mutants, where cytoplasmic condensations of *medial*- and *trans-*Golgi resident enzymes occurs, but the amount and localization of *cis*-Golgi proteins remains unaffected (Iwanami et al., 2016; Satoh et al., 2016).

### Rh1 is transported to Golgi units, but degraded in Syx5-deficient photoreceptors

**Fig. 5.**
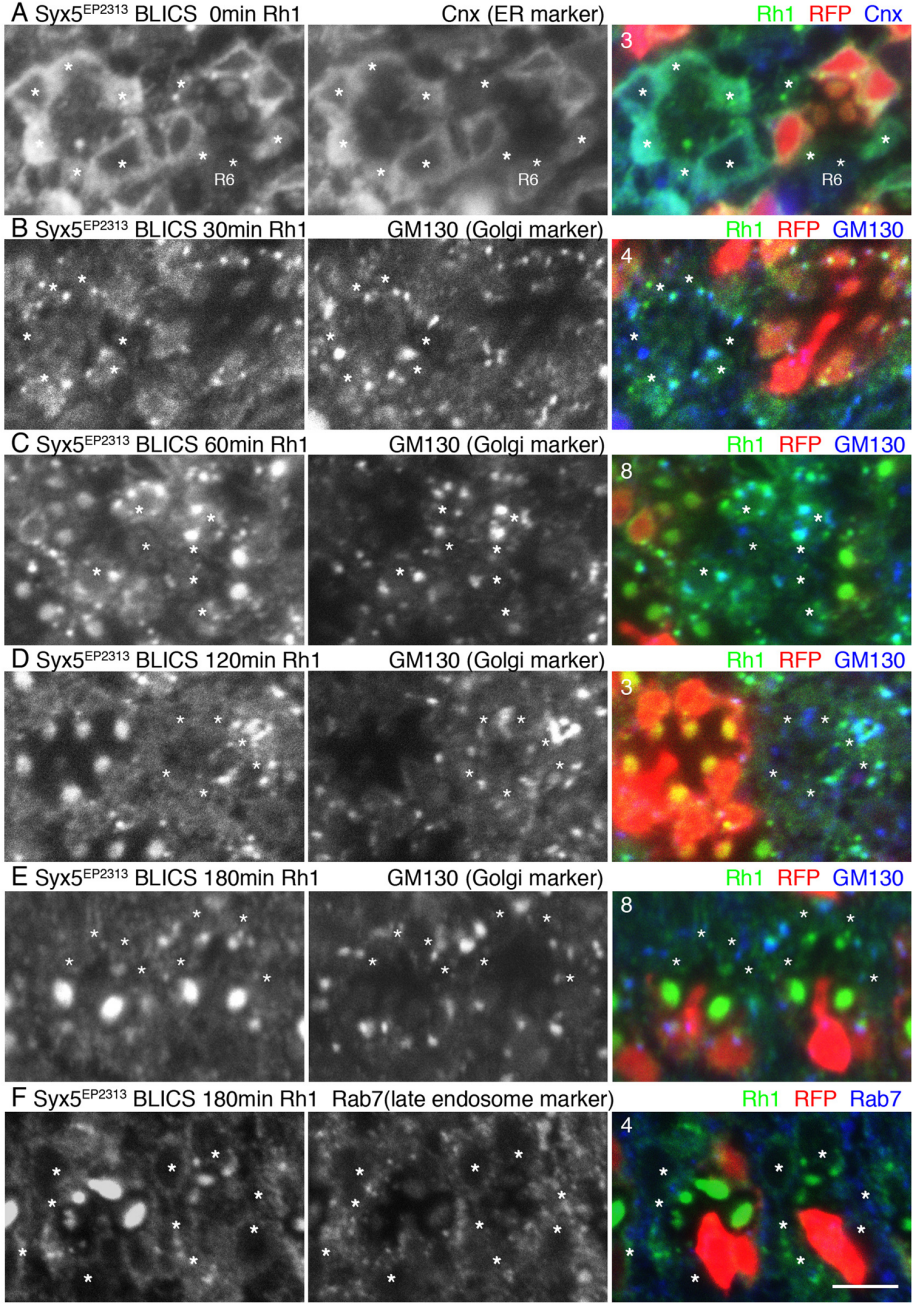
**Kinetics of Rh1 transport in *Syx5^EP2313^* mosaic retinas.** S*yx5^EP2313^* mosaic retinas were immunostained with antibodies described below. RFP (red) marks wild-type cells. Asterisks indicate *Syx5^EP2313^* homozygous photoreceptors. (A) Immunostaining before BLICS with anti-Rh1 (blue) and anti-Cnx (green) antibodies. Cnx is an ER marker. (B-E) Immunostaining of *Syx5^EP2313^* mosaic retinas 30 min (B), 60 min (C), 120 min (D), and 180 min (E) after BLICS, using anti-Rh1 (blue) and anti-GM130 (green) antibodies. GM130 is a Golgi marker. (F) Immunostaining of *Syx5^EP2313^* mosaic retinas with anti-Rh1 (blue) and anti-Rab7 (green) antibodies 180 min after BLICS. Rab7 is a late endosome marker. Scale bar: 5 μm. Numbers of the samples observed were shown in the top left corner of the composite images.

To determine the trafficking step at which Rh1 transport is inhibited in Syx5-deficient photoreceptors, we used the blue light-induced chromophore supply (BLICS) method (Ozaki et al., 1993; Satoh et al., 1997). Rh1 comprises an apoprotein and a chromophore, called opsin and *11-cis* retinal, respectively. In the absence of *11-cis* retinal, opsin accumulates in the ER and Rh1 is not transported to the Golgi units. Blue light-illumination photoisomerizes *all-trans* retinal to *11-cis* retinal, thereby inducing synchronous release of Rh1 from the ER into the secretory pathway. Prior to BLICS, levels of Rh1-apoprotein in the ER of most Syx5-deficient photoreceptors are similar to those in the wild-type cells, with a few exceptions in which Rh1-apoprotein was drastically reduced (an example shown in Fig. 5A). Similar levels of Rh1 accumulated in the Syx5-deficient and wild-type Golgi units 30 min after BLICS (Fig. 5B). Thus, after BLICS, Rh1 was transported to the Golgi units in Syx5-deficient photoreceptors with similar kinetics as compared to the wild-type cells (Fig. 5B). Rh1 staining in Syx5-deficient Golgi units was much stronger than that of the wild-type Golgi units, 60 min after BLICS. In the wild-type cells, some Rh1 signals also appeared in the rhabdomeres 60 min after BLICS, but no Rh1 signal on rhabdomeres was observed in Syx5-deficient cells (Fig. 5C). Three hours after BLICS, most of the BLICS-induced Rh1 reached the rhabdomeres; therefore, not many Rh1 signals were observed in the cytoplasm of the wild-type photoreceptors. However, only a small amount of Rh1 accumulated in Syx5-deficient rhabdomeres, 3 h after BLICS (Fig. 5E). Therefore, we observed that Rh1 transport was inhibited in Syx5-deficient photoreceptors. Surprisingly, Rh1 staining on Golgi units of Syx5-deficient photoreceptors 3 h after BLICS was much weaker as compared to the Rh1 signals observed 60 min after BLICS. In addition, Rh1 staining was not detected on the other organelle at the same time point (Fig. 5F), suggesting that most of the Rh1 is degraded 3 h after BLICS in Syx5-deficient photoreceptors. In other mutants associated with Rh1 transport defects, such as *Rab6* and *PIG* mutants, 3 h after BLICS there is a large number of late endosomal compartments and multi-vesicular bodies (MVBs) containing Rh1, indicating that Rh1 is degraded within MVBs, however, it takes more than 3 h to degrade Rh1 in the MVBs (Iwanami et al., 2016; Satoh et al., 2013). Therefore, we conclude that in Syx5-deficient photoreceptors, Rh1 is first transported to Golgi units and is then quickly degraded by MVBs-independent processes.

### TRP and Eys are degraded by ERAD in Syx5 deficient photoreceptors

**Fig. 6.**
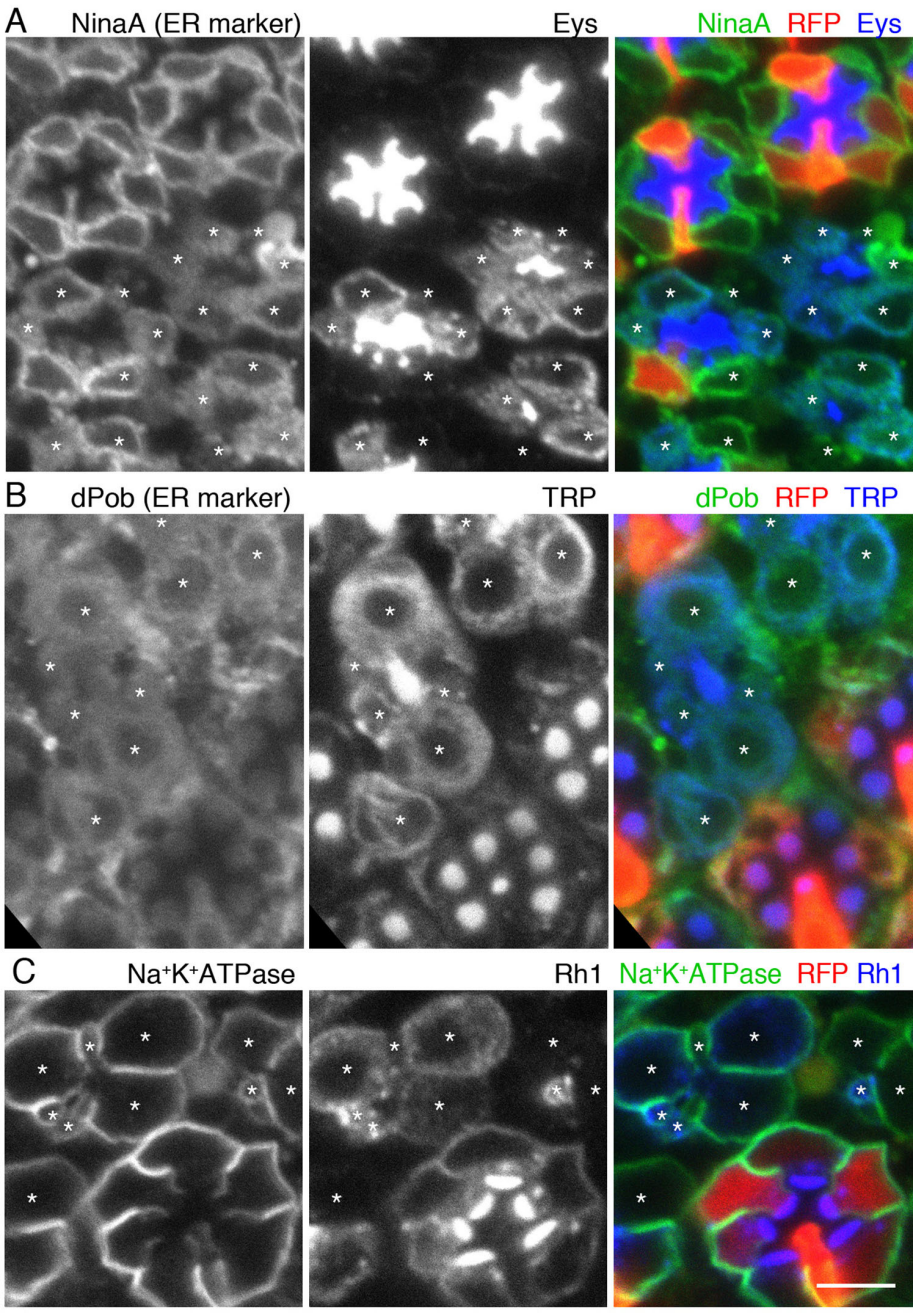
**TRP and Eys are degraded by ERAD system in Syx5-deficient photoreceptors.** *EDEM2 ^DG03809^* and *Syx5^EP2313^* mosaic retinas with a viable *EDEM1^EP1588^* homozygous mutation were immunostained by the indicated antibodies. RFP (red) marks *EDEM1^EP1588^* single mutant cells. Asterisks indicate *EDEM1^EP1588^*, *EDEM2 ^DG03809^* and *Syx5 ^EP2313^* triple deficient photoreceptors. (A) Anti-NinaA (green) and anti-Eys (blue) antibodies. (B) Anti-dPob (green) and anti-TRP (blue) antibodies. (C) Anti- Na^+^K^+^-ATPase (green) and anti-Rh1 (blue) antibodies. Scale bar: 5μm. Three samples were observed for each of stainings.

Besides MVBs, degradation of the membrane, luminal, and secretary proteins is also carried out by endoplasmic reticulum-associated protein degradation (ERAD). ERAD not only eliminates terminally misfolded or unassembled polypeptides, but also degrades surplus polypeptides, thereby regulating the level of enzymes and other molecules (Christianson and Ye, 2014; Ruggiano et al., 2014). Therefore, we investigated if the membrane and secretory proteins are degraded by ERAD in *Syx5* mutant photoreceptors. ER degradation enhancer, mannosidase alpha-like 1(EDEM1) and 2 (EDEM2) were reported to be involved in the degradation of misfolded P23H mutant rod opsin protein by ERAD in vertebrate (Kosmaoglou et al., 2009). Therefore, to inhibit *ERAD* in the *Syx5* mutant, we introduced mutations in *EDEM1* and EDEM2. As both *Syx5* and *EDEM2* genes are located on the same arm of the second chromosome, we constructed *Syx5 ^EP2313^, EDEM2 ^DG03809^* mosaic retina with *EDEM1^EP1588^* homozygous background, and we found abundant accumulation of TRP and Eys in *EDEM1^EP1588^*, *EDEM2 ^DG03809^*, and *Syx5 ^EP2313^* triple deficient photoreceptor cytoplasm (Fig. 6B,C). TRP and Eys co-localize with the ER markers, dPob and NinaA, respectively, suggesting that TRP and Eys accumulate on ER in triple deficient photoreceptors (Fig. 6A,B). However, Rh1 and Na^+^K^+^ATPase did not accumulate in EDEM1, 2, and Syx5 triple deficient photoreceptors (Fig. 6C). The difference in the accumulation of each protein must reflect the substrate specificity of EDEM1 and 2 (Słomińska-Wojewódzka and Sandvig, 2015). These results indicate that in Syx5-deficient photoreceptors, membrane and secretory proteins are degraded by ERAD.

### Massive accumulation of vesicles between Golgi cisternae and ER in *Syx5* mutant photoreceptors

**Fig. 7.**
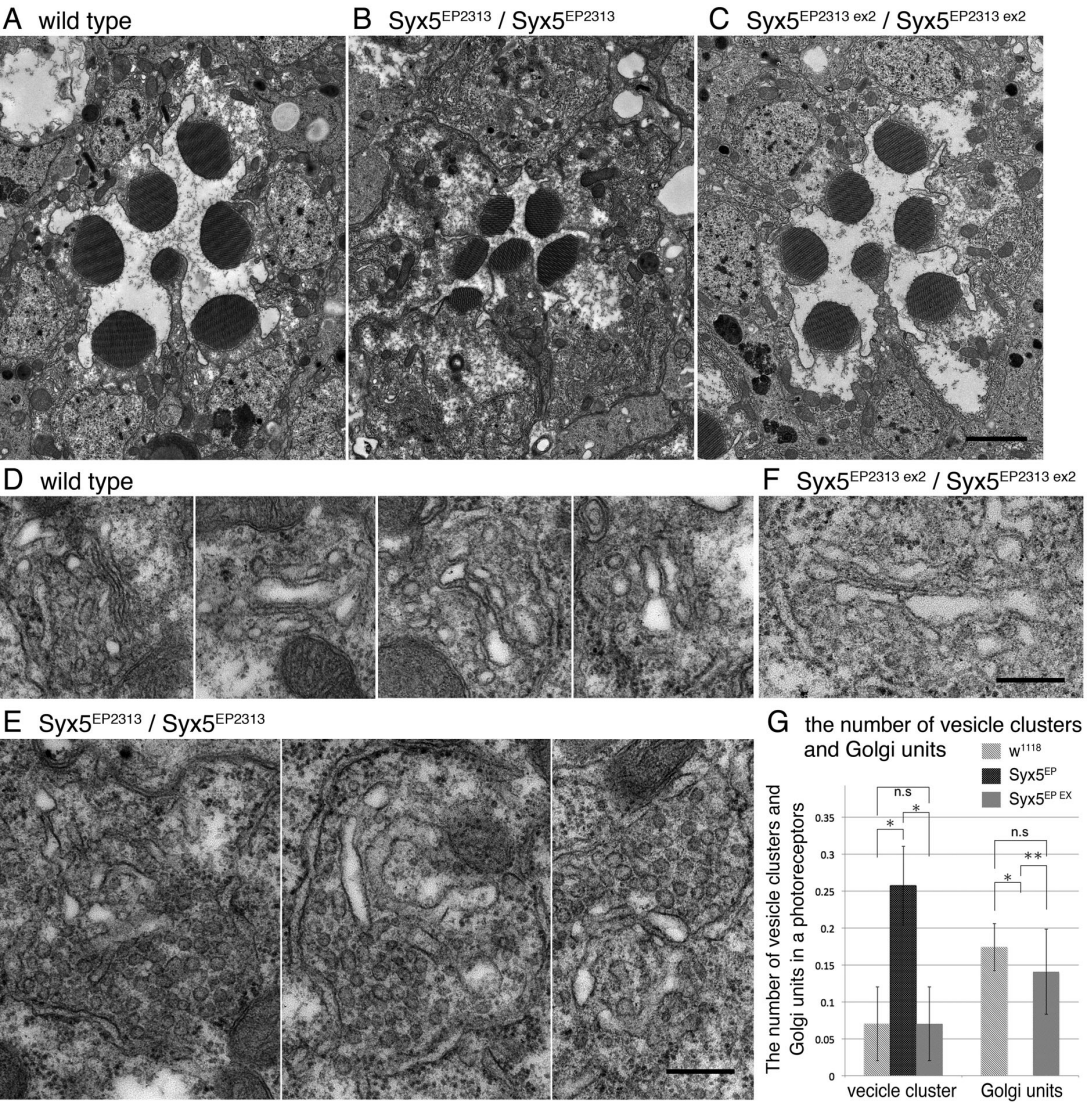
**Massive accumulation of vesicles between ER and Golgi units in Syx5-deficient photoreceptors.** Flies were reared in the dark and the retinal samples were fixed at late pupal stage. To avoid light-dependent Rh1 endocytosis, fixation was performed within 3 min of transferring the pupae to light. (A-C) Electron microscopy of the ommatidia with the following genotypes: wild-type (A), *Syx5^EP2313^* homozygous ommatidia (B), *Syx5^EP2313 ex2^* homozygous ommatidia (C). (D) Golgi units in wild-type photoreceptors. (E) Vesicle clusters in *Syx5^EP2313^* homozygous photoreceptors. (F) Golgi units in *Syx5^EP2313 ex2^* homozygous photoreceptors. (G) Quantifications of the number of the vesicle clusters and the Golgi units in the cross section of a photoreceptor. The wild-type: 0.07 (s.d.±0.05) vesicle clusters and 0.17 (s.d.±0.05) Golgi units, *Syx5^EP2313^/Syx5^EP2313^*: 0.25 (s.d.±0.02) vesicle clusters and no Golgi units, *Syx5^EP2313 ex2^/Syx5^EP2313 ex2^*: 0.07 (s.d.±0.05) vesicle clusters and 0.13 (s.d.±0.05) Golgi units. *P<0.05, **P<0.1 (Student's *t*-test between samples). Scale bar: 2 μm (A-C), 300 nm (D-F). Three samples were observed for each of genotypes.

To investigate the morphology of Syx5-deficient photoreceptors, we examined thin sections of late pupal S*yx5^EP2313^*, or *Syx5^EP2313 ex2^* mosaic retinas and *Syx5^EP2313^*, or *Syx5^EP2313 ex2^* whole-eye clones of newly eclosed flies under the electron microscope (Fig. 7). We observed that in wild-type ommatidium, seven round rhabdomeres were separated by a large IRS (Fig. 7A). However, in *Syx5^EP2313^* ommatidia, rhabdomeres were found to be much smaller and their IRSs were narrower than those in the wild-type, while *Syx5^EP2313 ex2^* ommatidia displayed smaller rhabdomeres in comparison to the wild-type, with a normal IRS (Fig. 7B,C).

In Syx5-deficient cells, late pupal stage ER appear healthy in contrast to the dilated ER in the photoreceptors expressing Rab1 dominant negative mutant protein (Satoh et al., 1997). Consistent with the previous report, the majority of *Syx5^EP2313^* mutant photoreceptors displayed larger numbers of ER membranes compared to that in the wild-type (Zhao et al., 2015). However, we found more notable abnormalities in Golgi units than ER. In the wild-type late pupal photoreceptors, each Golgi unit comprised of a couple of tightly stacked cisternae and some vesicles, while in *Syx5^EP2313^* mutant photoreceptors, we observed that the Golgi cisternae were often dilated and not stacked properly, along with a massive accumulation of vesicles between Golgi and ER (Fig.7D,E). These vesicles were electron dense and often connected to tubules, suggesting budding or fusion profiles; however, mitochondria and adherence junctions were found to be normal in *Syx5^EP2313^* mutant photoreceptors. Very few MVBs were found in wild-type and Syx5-deficient photoreceptors of the flies reared in the dark; there were 0.15 (s.d.±0.06) MVBs per cross-section of a wild-type photoreceptor and 0.05 (s.d.±0.02) MVBs per cross-section of a *Syx5^EP2313^* photoreceptor.

On comparing the Golgi units in wild-type and *Syx5* mutants, we observed that *Syx5^EP2313 ex2^* Golgi units contained more vesicles as compared to the wild-type Golgi units, though the overall Golgi structure in *Syx5^EP2313 ex2^* mutant was normal (Fig. 7F). To quantify the numbers of vesicle clusters and Golgi units in the wild-type, *Syx5^EP2313^* and *Syx5^EP2313 ex2^* photoreceptors, we defined more than eight gathering vesicles as a vesicle cluster and more than three cisternae stacks within a 300-nm area as a Golgi unit (Fig. 7G). Considering these parameters, a large number of vesicle clusters was detected in *Syx5^EP2313^* photoreceptors, but no Golgi units. However, the total sum of the vesicle clusters and Golgi units in *Syx5^EP2313^* and wild-type photoreceptors were same. Unlike *Syx5^EP2313^*, in *Syx5^EP2313 ex2^* photoreceptors the number of Golgi units and the vesicle clusters were similar to those found in the wild-type photoreceptors. We recently reported that Rab6-deficient Golgi cisternae appeared dilated, and accompanied by MBVs in comparison to the wild-type (Iwanami et al., 2016), however, unlike *Rab6* mutants, in *Syx5* mutants vesicle clusters were observed near the Golgi units and multi vesicular bodies (MVBs) were absent.

## DISCUSSION

In this study, we screened the failure of Rh1 accumulation on the rhabdomeres and identified Syx5 as an essential molecule for Rh1 transport. A detailed study of Rh1 kinetics was performed using BLICS, which indicated that Rh1-transport in Syx5-deficient cells is inhibited right after it exits ER. Rh1 reaches GM130-labeled *cis-*Golgi in a normal time course but remains there for a prolonged period when compared to that in the wild-type cells. The accumulated Rh1 on *cis-*Golgi is then most likely degraded by ERAD. Using electron microscopy, we observed massive vesicle accumulation between ER and Golgi units in Syx5-deficient photoreceptors. Massive accumulation of the COPII tethering factors, Rab1, and GM130 was also observed in the Golgi units of *Syx5* mutants. Therefore, we believe that in Syx5-deficient photoreceptors, the accumulated vesicles are most likely the COPII vesicles, thus, suggesting that Syx5 is involved in the fusion of COPII vesicles to *cis*-Golgi cisternae. These results are in agreement with the studies carried out in cultured cells (Kondylis and Rabouille, 2003, 2009; Mossessova et al., 2003; Xu and Hay, 2004).

Transport of membrane proteins and a secretory protein, Eys, to the three plasma membrane domains is inhibited in Syx5-deficient photoreceptors. This phenotype is distinct from the phenotype observed in the deficiency of Rab6, which is also reported to be involved in early Golgi transport (Girod et al., 1999; Martinez et al., 1994; White et al., 1999). In Rab6-deficient photoreceptors, the transport towards the two apical domains, rhabdomere, and the stalk are inhibited, but the transport towards the basolateral membrane domain is intact. Although Rh1 transport is inhibited within the Golgi units in both Syx5 and Rab6-deficient photoreceptors, the accumulated Rh1 is degraded differently in these mutants. In Rab6-deficient photoreceptors, Rh1 is degraded within the MVBs, which are formed from Golgi units directly (Iwanami et al., 2016). However, MVBs are not formed in Syx5-deficient photoreceptors, and Rh1 seems to be transported from the *cis-*Golgi area to ER by the retrograde transport – which functions normally in Syx5-deficient cells – and then degraded by ERAD.

**Fig. 8.**
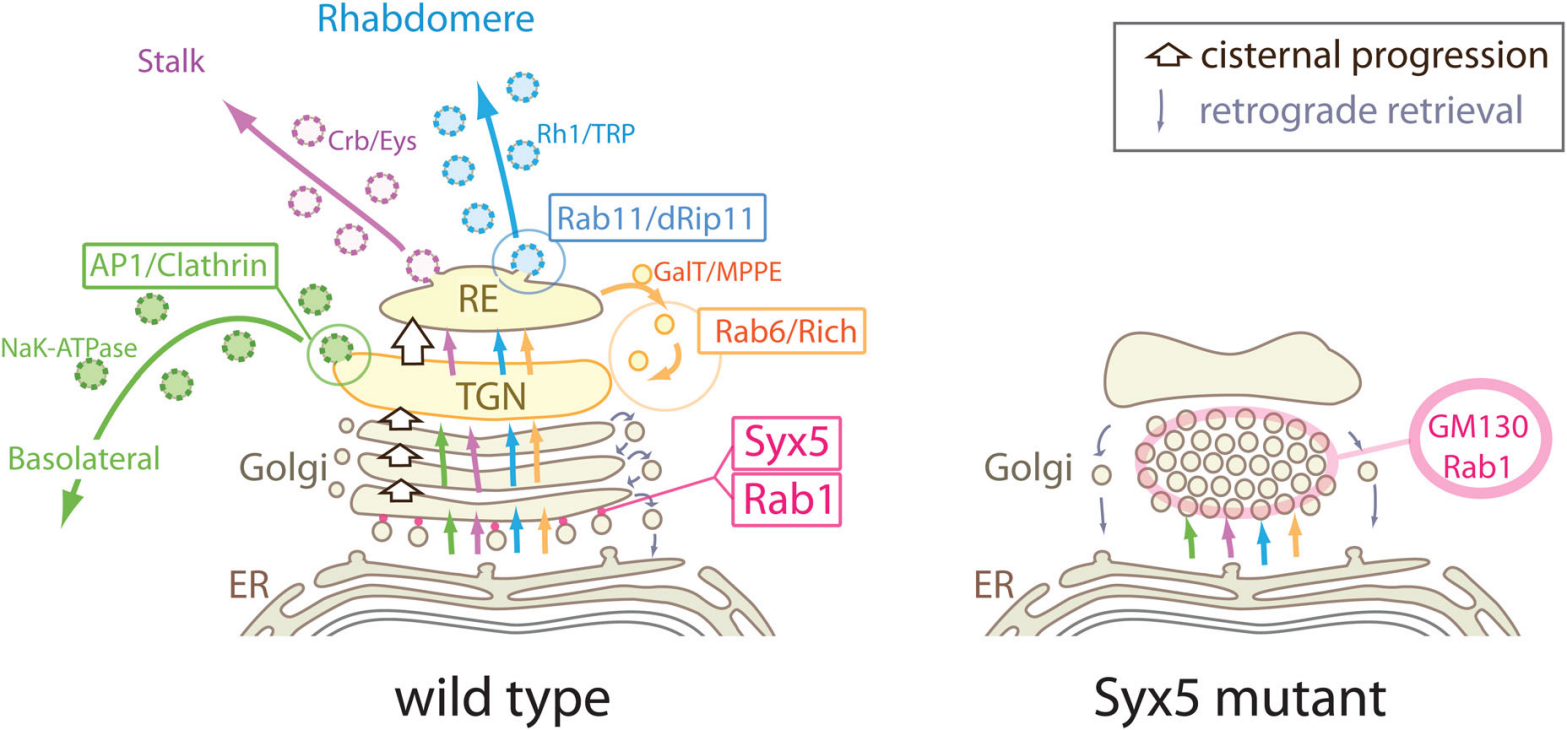
**Proposed model of Syx5 function and three polarized transport pathways.** Schematic diagram for polarized transport pathways in wild-type (left) and *Syx5^EP2313^* mutant (right) photoreceptors. Membrane proteins synthesized on the ER membrane are transported to *cis-*Golgi cisternae by Rab1 (Satoh et al., 1997), Syx5, and possibly by Sec22 (Zhao et al., 2015). Probably after Gos28 dependent processes (Rosenbaum et al., 2014), basolateral membrane proteins are sorted by AP1 at *trans-*Golgi/TGN (Satoh et al., 2013) and the two apical membrane proteins are transported together to the recycling endosome (RE) by Rab6 (Iwanami et al., 2016). There is another round of sorting for the two apical pathways at the RE. Rab11, dRip11, and MyoV then mediate the transport of post-Golgi vesicles carrying rhabdomere proteins (Li et al., 2007; Satoh et al., 2005). Finally, the exocyst complex tethers these post-Golgi vesicles to the base of the rhabdomeres (Beronja et al., 2005).

We find that Syx5 and Rab6 function at different stages of the transport, which is further corroborated by difference in localization of the two proteins, and differential distribution of Golgi resident proteins in Syx5- and Rab6-deficient photoreceptors. We have summarized Golgi morphologies and distribution of Golgi resident proteins in wild-type and Syx5-deficient photoreceptors in Fig. 8. Syx5 localizes to *cis*-Golgi together with Rab1, extending slightly further towards the *cis*-side as compared to GM130 localization. In Syx5-deficient photoreceptors, *cis-*Golgi markers, Rab1 and GM130 display increased accumulation, whereas *medial-* or *trans-*Golgi resident membrane proteins, MPPE and GalT::CFP, respectively, disappeared from the Golgi units. In addition, a massive accumulation of vesicles between ER, Golgi cisternal membranes and cisternal dilation were observed in Syx5-deficient photoreceptors, which may be attributed to the localization of Rab1 and GM130 on these vesicles. Reduced numbers of Golgi cisternal membrane in Syx5-deficient photoreceptors might explain the lack of MPPE and GalT::CFP, which is due to lack of *medial-* or *trans-*cisternae in the Golgi units of Syx5-deficient photoreceptors. These results indicate that Syx5 functions in early Golgi trafficking, probably in the fusion of COPII vesicle to *cis-*Golgi cisternae.

In contrast to Syx5, Rab6 localizes to GalT::CFP-positive TGN or *trans*-Golgi cisternae extending further towards the *trans*-side, to Golgi-associated Rab11-positive recycling endosome (Iwanami et al., 2016). In Rab6 deficient photoreceptors, cisternal membrane of Golgi units are dilated, but overall structures of Golgi units remain unaffected; no accumulation of vesicles or reduction of cisternae is observed (Iwanami et al., 2016). Similarly, GM130 localizes normally on Golgi units whereas MPPE and GalT::CFP are absent in the Golgi units but are instead observed as loose cytoplasmic condensation in Rab6 deficient photoreceptors (Satoh et al., 2016). Localizations of Golgi resident proteins in Rab6-deficient photoreceptors differ from those in Syx5-deficient photoreceptors, indicating that Syx5 functions at an earlier step in intra-Golgi transport, in comparison to Rab6.

Fly photoreceptors are an excellent model system to investigate the mechanism of membrane transport, especially polarized transports. This work gives the foundation of the phenotypic analysis of the inhibition on the step before polarized transports. It would be quite useful for the future investigations of the polarized transports.

## MATERIAL AND METHODS

### *Drosophila* stocks and genetics

Flies were grown at 18–25°C on standard cornmeal-glucose-agar-yeast medium, unless indicated otherwise. For live-imaging and immunostaining in the Bruin flies, the following tester lines were used: *y w; P3RFP FRT40A/SM1; Rh1Arr2GFP eye-FLP/TM6B* were used for live-imaging, while *y w ey-FLP; P3RFP FRT40A / SM1* were used for immunostaining (Satoh et al., 2013). For P-element excision, *wg^SP-1^/CyO; ry, Sb, Delta2-3/TM6B* (Kyoto stock number, 107139) lines was crossed to *Syx5^EP2313^, FRT40A/CyO*, or *Syx5^EY07901^, FRT40A/CyO* line. Then *Syx5^EP2313^, FRT40A/CyO; ry, Sb, Delta2-3/+* males were crossed with the females of tester lines for live-imaging. The mosaic eye phenotypes of male progenies with the genotype, *Syx5 ^EP2313 ex^, FRT40A/CyO; Rh1Arr2GFP eye-FLP* or *Syx5 ^EY07901 ex^, FRT40A/CyO; Rh1Arr2GFP eye-FLP* were observed using confocal microscope. Some of them were crossed to *Sco/Cyo* and a couple of excision lines, *Syx5 ^EP2313 ex^, FRT40A/CyO* or *Syx5 ^EY07901 ex^ FRT40A/CyO* were established. For the analysis of *EDEM1^EP1588^*, *EDEM2 ^DG03809^* and *Syx5 ^EP2313^* triple deficient photoreceptors: *w, EDEM1 ^EP1588^*; *EDEM2 ^DG03809^, Syx5 ^EP2313^, FRT40A* were crossed with *y w EDEM1 ^EP1588^; P3RFP FRT40A/SM1* and *ey-FLP/+*.

The following fly stocks were used: *Rh1-Gal4*, *GMR-Gal4*, *EGUF40A* (Bloomington stock number, 5250), *EDEM1^EP1588^* (Bloomington stock number, 17011), *EDEM2 ^DG03809^* (Bloomington stock number, 21030) *UAS-CFP::GalT* (Satoh et al., 2005) and *UAS-Syx5::Myc* (from Dr Burke, Monash University).

### Live-imaging of fluorescent proteins expressed in photoreceptors

Fluorescent proteins expressed in the photoreceptors were imaged by a water-immersion technique as described previously (Satoh et al., 2013).

### Immunohistochemistry

The samples were fixed and stained as per the protocol described previously (Satoh and Ready, 2005). Primary anti-sera used were: rabbit anti-Rh1 (1:1000) (Satoh et al., 2005), chicken anti-Rh1 (1:1000) (Satoh et al., 2013), Rat anti-dPob (1:100) (Satoh et al., 2015), Rabbit anti-NinaA (1:300) (a gift from Dr Zuker, Columbia University), mouse monoclonal anti-KDEL (1:100) (Assay Designs, Ann Arbor, MI, USA, No.SPA-827), Rabbit anti-Cnx (1:100) (Iwanami et al., 2016), Rabbit anti-GM130 (1:300) (Abcam, Cambridge, UK, No. ab30637), mouse anti-Rab1 (1:250) (Satoh et al., 2005), rabbit anti-MPPE (1:500) (a gift from Dr Han, Southeast University), chicken anti-GFP (1:1000) (Chemicon International Inc., Billerica, MA, USA, No.AB16901), rat monoclonal anti-p120 (1:12) (a gift from Dr Goto, Rikkyo University) (Yamamoto-Hino et al., 2012), Guinea Pig anti-Rab6 (1:300) (Iwanami et al., 2016), mouse monoclonal anti-Myc (1:12) (DSHB, IA, USA), rabbit anti-Myc (1:300) (MBL, Nagoya, Japan, No.562), mouse monoclonal anti Na^+^K^+^ATPase alpha subunit (1: 500 ascites) (DSHB, IA, USA), rabbit anti-TRP (1:1000) (a gift from Dr Montell, Johns Hopkins University), Rabbit anti-Rab7 (1:1000) (a gift from Dr Nakamura, Kumamoto University), mouse monoclonal anti-Eys (1:20 supernatant) (DSHB, IA, USA), Rat monoclonal anti-DECad (1:20 supernatant) (DSHB, IA, USA), mouse monoclonal anti-Chp (1:20 supernatant) (DSHB, IA, USA) and rabbit anti-Chc (1:500) (a gift from Dr Kametaka, Nagoya University).

Secondary antibodies used were: anti-mouse, -rabbit, -rat, and/or -chicken labeled with Alexa Fluor 488, 568, or 647 (1:300) (Life Technologies, Carlsbad, CA, USA). Samples were analyzed and images were recorded using a FV1000 confocal microscope (60X, 1.42NA lens, Olympus, Tokyo, Japan).

### Blue light-induced chromophore supply (BLICS)

Newly eclosed flies fed on carotenoid-deprived food (1% agar, 10% dry-yeast, 10% sucrose, 0.02% cholesterol, 0.5% propionate and 0.05% methyl 4-hydroxybenzoate) were switched to carotenoid-deprived food supplemented with crystalline *all-trans*-retinal (Sigma, St. Louis, MO, USA) in the dark. After one or two nights in the dark, flies were irradiated with a 405 nm diode laser module at 30 mW for 20 min (Pepaless, Hyogo) to isomerize the *all-trans* retinal to 11-*cis*-form and initiate Rh1 exit from the ER.

### Electron microscopy

Electron microscopy was performed as described previously (Satoh et al., 1997). Samples were observed under JEM1400 electron microscope (JEOL, Tokyo, Japan), and montage images were captured by a CCD camera system (JEOL, Tokyo, Japan).
